# Code-mixing unveiled: Enhancing the hate speech detection in Arabic dialect tweets using machine learning models

**DOI:** 10.1371/journal.pone.0305657

**Published:** 2024-07-17

**Authors:** Ali Alhazmi, Rohana Mahmud, Norisma Idris, Mohamed Elhag Mohamed Abo, Christopher Ifeanyi Eke

**Affiliations:** 1 Faculty of Computer Science and Information Technology, Universiti Malaya, Kuala Lumpur, Malaysia; 2 Department of Computer Science, College of Engineering and Computer Science, Jazan University, Jazan, Saudi Arabia; 3 Faculty of Computing, Department of Computer Science, Federal University of Lafia, Lafia, Nasarawa State, Nigeria; Zayed University, UNITED ARAB EMIRATES

## Abstract

Technological developments over the past few decades have changed the way people communicate, with platforms like social media and blogs becoming vital channels for international conversation. Even though hate speech is vigorously suppressed on social media, it is still a concern that needs to be constantly recognized and observed. The Arabic language poses particular difficulties in the detection of hate speech, despite the considerable efforts made in this area for English-language social media content. Arabic calls for particular consideration when it comes to hate speech detection because of its many dialects and linguistic nuances. Another degree of complication is added by the widespread practice of "code-mixing," in which users merge various languages smoothly. Recognizing this research vacuum, the study aims to close it by examining how well machine learning models containing variation features can detect hate speech, especially when it comes to Arabic tweets featuring code-mixing. Therefore, the objective of this study is to assess and compare the effectiveness of different features and machine learning models for hate speech detection on Arabic hate speech and code-mixing hate speech datasets. To achieve the objectives, the methodology used includes data collection, data pre-processing, feature extraction, the construction of classification models, and the evaluation of the constructed classification models. The findings from the analysis revealed that the TF-IDF feature, when employed with the SGD model, attained the highest accuracy, reaching 98.21%. Subsequently, these results were contrasted with outcomes from three existing studies, and the proposed method outperformed them, underscoring the significance of the proposed method. Consequently, our study carries practical implications and serves as a foundational exploration in the realm of automated hate speech detection in text.

## 1. Introduction

In recent decades, technological advancements have transformed the way people communicate in society. Platforms like social media and blogs have become essential channels for individuals worldwide to express their viewpoints, feelings, and thoughts on various subjects. Nevertheless, these platforms have witnessed a significant rise in the spreading of prejudiced, aggressive, and hate-filled content. Hate speech (HS), in broad terms, involves the utilization of language that specifically singles out particular groups according to attributes like their ethnicity, religion, or gender [1]. HS in the digital realm is a multifaceted notion encompassing an extensive array of target manifestations, and closely associated notions [2,3]. While there is not a single, accepted definition of HS, there is agreement among scholars and service providers to define it as language that initiates attacks against a person or a group according to attributes like race, ethnicity, gender, sexual orientation, nationality, or religion [3]. This form of prejudiced and hateful expression can wield a detrimental influence on the community, as it jeopardizes the principles of cohabitation and solidarity. Furthermore, it is clear that HS shares a profound correlation with real-world hate-related crimes [4], amplifying the perils linked to being subjected to such hateful language and abusive expressions.

Twitter actively promotes the reporting of any HS that contravenes its hateful conduct policy, enabling measures such as user suspension or tweet removal to be taken [5]. Even with the substantial initiatives undertaken by social media platforms to combat HS, it continues to jeopardize online communities, persisting across various platforms. Given that HS can lead to severe physical or psychological harm, there exists a pressing requirement to identify and mitigate the posting of such content on social networking sites. Identification and continuous monitoring of HS represent the primary strides in establishing effective countermeasures against and addressing the proliferation of offensive information across social networking sites. Facebook and microblog sites have faced criticism for their perceived inadequate efforts in mitigating HS on their platforms, which has led to the enforcement of policies prohibiting the utilization of their sites to publicly criticize someone based on attributes like race, ethnic group, or religion [6]. Social media platforms that lack an automated system for detecting and filtering hateful tweets, primarily relying on manual moderation, encounter the challenge of extending the visibility of offensive content. As per the findings of [7], the longer offensive information stays available on the internet, the more severe the harm and repercussions it imposes.

Consequently, it has become critical to develop technologies capable of automatically detecting HS. Researchers in computer science and machine learning have created algorithms that can automatically detect HS on social media networks. These algorithms can help reduce the spread of such harmful information on these sites. Most of these algorithms concentrated on detecting HS written in English [8–12]. For example, the authors of [8] proposed HS detection using automated text categorization methods. In this research, three different techniques for feature engineering and eight machine learning algorithms were compared in order to accurately classify HS. In another study, [9] proposed some solutions for online HS detection using SVM and NB algorithms. Beside English, researchers have also applied machine learning (ML) algorithms to detect HS in various languages, including Spanish, German, and Italian [13–15]. In some cases, there exists code-mix in hate speech expressions in social media. Code-mixing is a frequent occurrence in which speakers combine two or more languages in one utterance or discussion. For tasks involving natural language processing (NLP), including hate speech detection, this presents major challenges. In code-mixed languages, machine learning algorithms play a major role in translating and identification of hate speech. The complexity of code-mixing may be handled and precise transcription and detection of hate speech in other languages outside English can be achieved by utilizing cutting-edge methods in language identification, translation, embedding, tokenization, and model training. The wide range of languages of social media and other communication platforms requires the development of strong natural language processing (NLP) solutions, which is made possible by these contributions.

However, the realm of detecting HS in Arabic content remains in its early stages [16]. This can be attributed to the intricate and multifaceted characteristics of the Arabic language, which encompasses a diverse range of dialects, as well as challenges at the orthographic, morphological, and syntactic levels [17]. These intricacies collectively contribute to the considerable complexity of detecting HS within Arabic content. Code-mixing, also referred to as language alternation or language switching, occurs when speakers use multiple languages or language varieties within a single conversation or sentence. Moreover, code-mixing occurs frequently in the informal written and spoken communication of social media users [18]. According to [19], code mixing in communication is not only the amalgamation of codes from distinct languages but necessitates a profound comprehension of both languages and the intercultural norms inherent in them. Thus, the presence of code-mixing makes it harder to accurately detect HS on Arabic tweets.

The main objective of this study is to investigate the use and effects of machine learning techniques in translating and identifying hate speech in Arabic, especially in code-mixed language scenarios. This can be done by assessing and comparing the effectiveness of different features and machine learning models for HS detection on the Arabic dialectics HS dataset and the code-mixing HS dataset. In this study, we used machine learning models to evaluate whether code-mixing can improve the effectiveness of HS identification in tweets written in the Arabic dialect. This work has made the following significant contributions:

1. Introduction of a Large Transliterated Arabic Corpus: The study introduced a large transliterated Arabic corpus of tweets collected from Twitter. This corpus was created to capture the nuances of code-mixed Arabic dialects, frequently found on social media platforms such as Twitter. We created this corpus to offer researchers and practitioners a helpful resource for studying and tackling HS in Arabic dialects.
2. Application of a Hybrid Annotation Approach: The authors employed a hybrid annotation approach, which involved mixing manual and machine methods, to annotate the dataset. This strategy was selected to capitalize on the advantages of both manual and machine annotation techniques and to tackle the difficulties related to annotating large datasets.
3. Comparative Analysis of Machine Learning Algorithms: The research presented a comparative analysis of the results of diverse supervised machine learning algorithms for identifying HS content within the transliterated Arabic corpus. We evaluated the performance of several popular machine learning algorithms, including Logistic Regression (LR), Naive Bayes (NB), Random Forest (RF), K-Nearest Neighbors (KNN), and Stochastic Gradient Descent (SGD). By comparing the results of these algorithms, the HS identification model attained the highest accuracy of 98.21% when employed with the SGD model.

The remainder of the study’s section is provided as follows: Section 2 outlines the literature review on HS identification. In Section 3, we explained the proposed methodology, encompassing dataset preparation, feature extraction and selection, and the construction of the classification model. Section 4 provides the experimental settings, including the evaluation metrics. Section 5 presents the results and discussions. We conclude the research in Section 6, followed by future work.

## 2. Literature review

The prevalence of HS has become a progressively significant concern in both digital and physical spaces, manifesting as prejudiced language and expressions that target specific people or groups based on attributes like gender, color, religion, or ethnicity [20]. HS identification on social network platforms has garnered increased focus from academics in recent times. Whether employing traditional machine learning techniques or deep neural networks, most preceding studies have approached the problem as a supervised classification problem [1,3]. Arabic language diversity is one of the most notable challenges in detecting HS. One can encounter various interpretations of a single term in Arabic due to the diversity of dialects. Constructing accurate HS detection models across various dialects is thus rendered challenging. Several studies, however, have demonstrated that models trained on a single dialect cannot be modified to function with other dialects [21,22]. Transfer learning has also been employed by researchers to enhance the efficacy of HS identification models in the Arabic language [23].

Several studies have been conducted to address HS detection using machine learning techniques and various features. For instance, Warner and Hirschberg [24] presented some of the earliest research on the problem of HS detection. Waseem and Hovey [25] proposed an approach based on machine learning for identifying hate speech on Twitter. The authors used a dataset consisting of 16,914 tweets. From each tweet, they extracted features in the form of unigrams, bigrams, trigrams, and fourgrams. Furthermore, they derived additional features, such as user gender and location, from user descriptions. They employed a logistic regression classifier and 10-fold cross-validation, and the best result obtained was 73.89 with a combination of bi- to four-grams and gender features. However, the study only considered English tweet data but failed to consider the code-mixing dataset, which is one of the major problems in social media data. Also, the study did not identify the research gap at the classification level.

In another study, [26] conducted a study aimed at categorizing sensitive topics found in social media comments and posts. The authors collected 7,500 comments from YouTube for the study. They employed the unigram technique in conjunction with the term frequency-inverse document frequency feature extraction method to create a numerical feature matrix. These generated features were then input into four distinct machine learning algorithms. Findings from the experiment revealed that the rule-based model recorded outstanding performance compared to other models, including decision tree, support vector machine, and Naïve Bayes models, achieving an accuracy rate of 73%. However, a notable limitation of rule-based modeling approaches is their restricted capacity to represent intricate patterns and correlations present in the data. The complexities seen in real-world datasets may be too complex for rule-based models, which depend on explicitly established rules or heuristics. Therefore, these models may struggle to effectively generalize to previously unobserved data. Moreover, the study failed to identify the research gap in terms of preprocessing techniques in handling the code-mixing expression in the dataset.

In a similar vein, [27] employed a machine learning method to categorize HS on social networking platforms. They used a public dataset containing 14509 English tweets. The author utilized the character-grams technique with 4 grams to generate numerical features. The author then fed these numeric features into an SVM classifier. The outcomes showed a maximum accuracy rate of 78%. However, the study only focused on the English dataset and did not consider hate speech data that was composed in other languages, especially the Arabic language, which is the focus of this study.

To evaluate the performance of word embedding features, [10] examined word embeddings as features. The authors collected 951,736 comments from the Yahoo Finance website. In order to detect abusive language in user comments, they experimented with low-dimensional text embeddings using paragraph2vec, and the experimental analysis shows that paragraph2vec outperformed BOW representations in terms of the AUC-ROC curve. However, a notable issue with low-dimensional text embeddings is the possible loss of contextual information during feature extraction.

In another study, the authors [6] collected 24,802 posts from Twitter. They used unigram, bigram, and trigram features, each weighted by its TF-IDF. Logistic regression with L1 regularization was used to reduce the dimensionality of the data. They used logistics regression, naive bayes, decision trees, random forests, and linear SVMs. The best model exhibits an overall precision of 0.91, a recall of 0.90, and an F1 score of 0.90. However, the study did not identify research gaps in the methodology of the reviewed literature at the preprocessing and classification levels.

In recent times, certain research investigations have also assessed the efficacy of deep learning (DL) methodologies, a subset of machine learning, for HS identification. For instance, a DL method was proposed by the authors [28] to automatically detect HS in the Saudi Twittersphere. They built a dataset containing a total of 9316 tweets classified as hateful, abusive, or normal. The features are represented as word embeddings using the Word2Vec architecture. They evaluated four different models: CNN, GRU, CNN+GRU, and BERT. The findings showed that CNN had superior performance compared to other models, achieving an F1-score of 0.79 and an AUROC of 0.89. Moreover, the results showed that BERT did not demonstrate any improvement compared to the baselines and the other models that were evaluated. Even though a promising result was obtained, word2vec frequently fails when dealing with words that are polysemic (meaning they have numerous meanings) or whose meanings are highly contextualized.

Moreover, researchers In [29], researchers investigated the impact of merging gated recurrent units with customized features for detecting religious hostility in Arabic tweets. 10,000 annotated tweets from public Arabic tweets were purposefully compiled by the researchers to detect religious HS. The researchers used deep learning models with handcrafted features to improve hate speech detection methods. The method employed GRU to learn tweet sequences and handcrafted features to capture religious hate speech’s linguistic and semantic characteristics. Additionally, they compiled three public Arabic lexicons containing terms frequently encountered in conversations related to religion, along with scores indicating their sentiment and intensity. The findings reveal that GRU-based RNNs, when combined with pre-trained word embeddings, showed superior performance compared to other lexicon-based and n-gram-based classifiers. However, the study did not consider the impact of code-mixing in detecting hate speech in Arabic dialectics.

The authors [30] used a public dataset of 15,050 posts from Arabic celebrity videos. They used Ara-Vec pre-trained word embedding for feature representation. The authors employed CNN, Bi-LSTM, attention mechanisms, and the combination of CNN and LSTM models to classify the data. The integration of CNN and LSTM achieved the best results, with a recall of 83.46%. The results also showed that CNN alone can only learn local features from word n-grams, whereas the CNN-LSTM combined model can also learn global features and long-term dependencies due to the LSTM layer. However, one of the drawbacks of pre-trained models is that they can function well on a variety of tasks as a result, although they might not be tailored for certain applications or domains. Consequently, when used for jobs in specialized disciplines, pretrained models may perform less well because they are unable to catch the subtleties or terminology unique to a certain domain.

Some authors conducted a comparative examination to assess the effectiveness of both ML and DL on Arabic tweets for HS detection. For instance, [31] constructed an Arabic HS dataset from Facebook, Instagram, YouTube, and Twitter. There were 20,000 postings, tweets, and comments. A total of 12 ML and DL algorithms were evaluated. RNN delivered the highest accuracy of 98.7%. The study only focused on the comparative analysis of various learning models for hate speech detection but was unable to consider the effect of code-mixing performance in Arabic dialectics.

The findings from previous research revealed that a wide range of scholars globally are focusing on HS identification in Twitter data, comprising various languages, including Hindi, Arabic, and English. To the extent of our understanding, none of the research has compared the performance of various features and machine learning algorithms on the standard Arabic dialectics’ dataset and the code-mixing dataset, which will be an anchor and basis for this study. Moreover, the existing studies did not identify research gaps in the methodology of the reviewed literature at the preprocessing and classification levels. Building on the recent published literature review on hate speech detection in Arabic Twitter data [32], this research seeks to investigate the use and effects of machine learning techniques in translating and identifying hate speech in Arabic dialectic language, especially in code-mixed language scenarios. This can be done by demonstrating if the approach performs well on HS datasets by comparing three feature engineering, five ML classifiers on standard Arabic tweets, and code-mixing tweets on HS datasets.

## 3. Materials and methods

**Fig 1:**
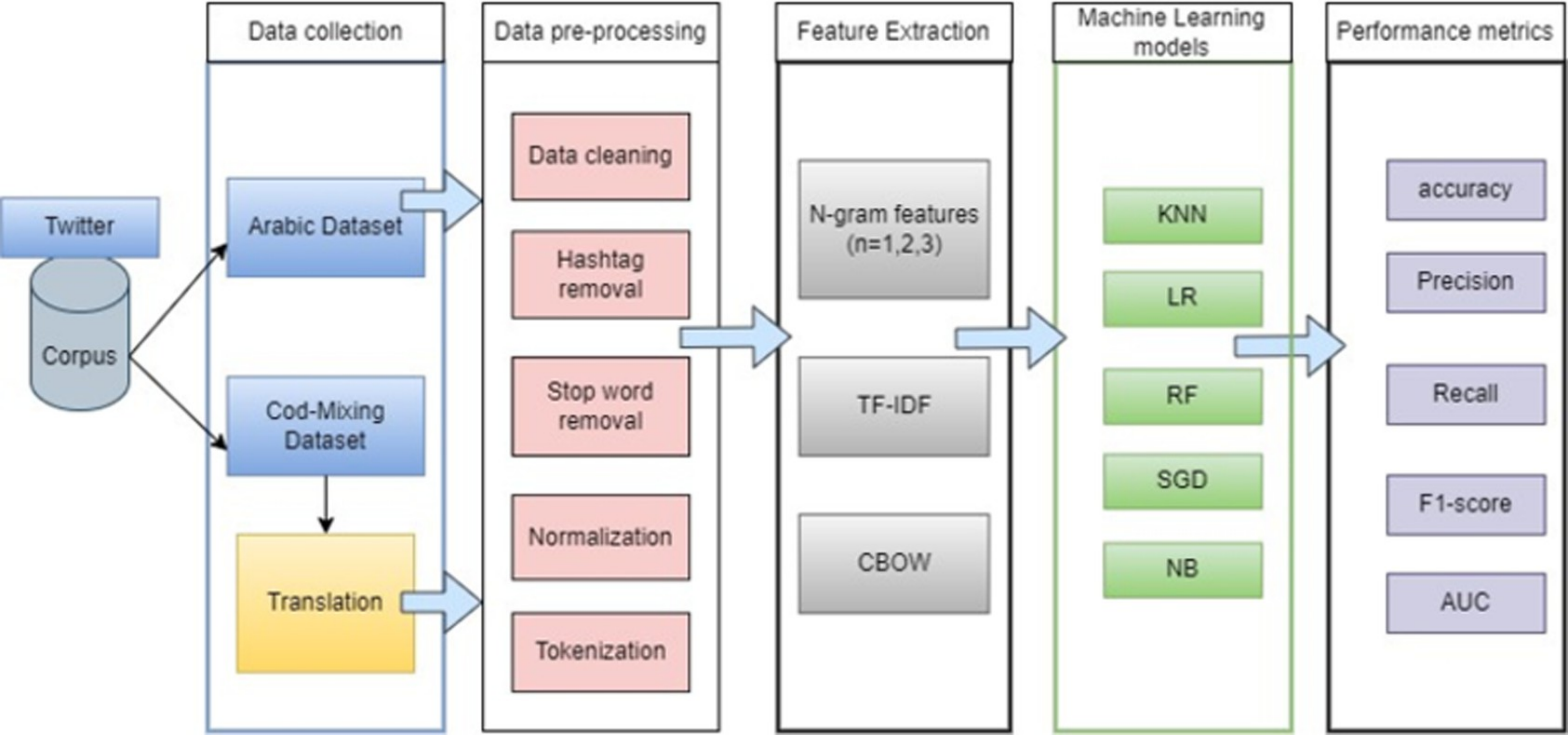
Arabic hate speech detection framework.

This segment provides the method proposed in this study: “Code-Mixing Unveiled: Enhancing the Hate Speech Detection in Arabic Dialect Tweets Using Machine Learning Models” that we used for tweet classification into two groups: "hate speech" and "not hate speech." The whole research procedure is displayed in Fig 1, which illustrates the six essential phases that make up the research methodology, including data collection, preprocessing, feature extraction, building the classifiers, and evaluating the classifiers. The subsection that follows details each phase of the framework.

### 3.1 Data collection and description

**Fig 2:**
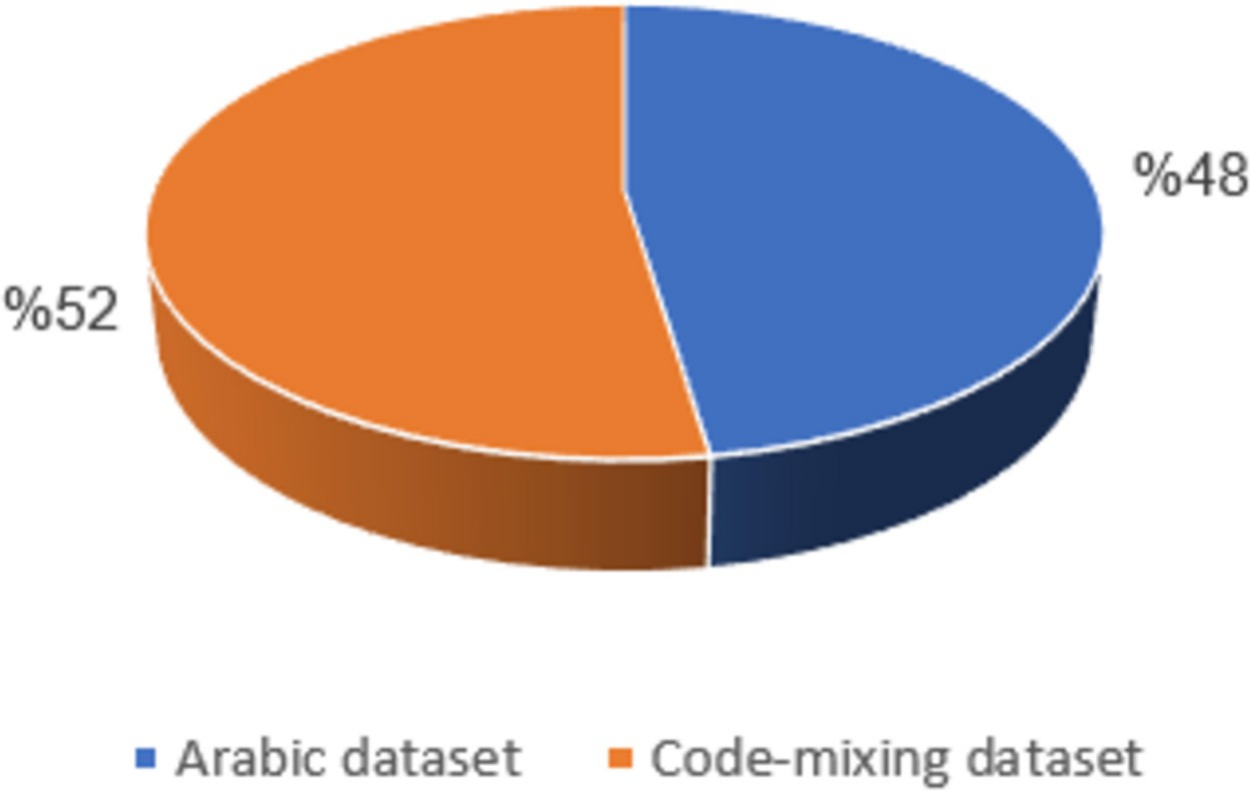
Visualization of dataset sizes.

The creation of a novel dataset containing several types of HS, including those pertaining to race, religion, and ideology, was the focus of this study. Data collection was performed by employing a standard Twitter API on Arabic tweets and code-mixing datasets [33]. Twitter stands out as a prominent microblogging platform, allowing users to share thoughts, emotions, and other conversations among themselves. A notable import of Twitter data lies in its abundant availability, given the continuous stream of user-generated messages. The Twitter API serves as a crucial link between users and Twitter servers, facilitating easy access to archived tweets. Leveraging the API enables the retrieval of tweets from the public domain, each containing comprehensive user information, such as identification of users, URLs, usernames, account particulars, and the essential content of tweets. This text feature, rich in emotional, behavioral, and informational content, is vital for analysis [34]. The collected information forms the foundation for constructing a set of features crucial for the efficient categorization of Twitter data [34,35]. Moreover, this data has served as instrumental in developing a suggested model by identifying significant features for machine learning model training. This model aims to differentiate between expressions of HS and not HS in Arabic tweets. Data collection was carried out for a duration of three months, specifically from April 2023 to June 2023. This was done via a combination of keyword, thread-based searches, and profile-based search approaches, as shown in Table 2. A total of 120 terms, including various versions, were included in the search query for the keyword-based approach. These terms referred to groups or individuals frequently targeted by HS, such as regional references like ("Gizani", "جيزاني") and its various forms (“جيzاني” , “جيز3ني”, “gيزاني” ), which were used to identify tweets containing code-mixing concerning regional HS. To conduct a thread-based search, we have incorporated hashtags that are related to contentious subjects that are deemed essential markers for hateful speech. Throughout the data-gathering phase, we kept an eye on Twitter trends and designated ten hashtags for information retrieval. Given that hateful tweets are usually less common than regular tweets, we expanded our dataset and improved the representation of the hate class by incorporating the most impactful terms from a lexicon of religious hate terms released by Albadi et al. [36]. We gathered exclusively original Arabic tweets for all queries, excluding retweets and non-Arabic tweets. In all, we obtained 200,000 Twitter data, of which we sampled 23k tweets for annotation, as can be shown in Fig 2. Examples of keywords utilized to obtain data are depicted in Table 1, whereas the summary of the dataset description is shown in Table 2.

**Table 1:** Example of keywords for data extraction.

Hate Speech category	keywords
**Racist**	جيزاني، صلبي، طرش بحر, بقايا حجاج
**Sexism**	نسويات، مكانها المطبخ
**Offensive**	List of keywords known as offensive toward groups or individuals.

**Table 2:** Dataset description summary.

Data source	Twitter
Data collection method	keyword, thread-based searches, and profile-based searches approach
Tweets language	Arabic
Class of data	Hate speech and non-hate speech
Data collection period	April 2023 to June 2023
Annotation approach	Expert opinion
Arabic dataset volume	Count of Offensive: 3867 Count of Not Offensive: 7364 Total: 11,231
Code-mixing dataset volume	Count of Offensive: 7326 Count of Not Offensive: 5030 Total: 12,356
The total volume of the dataset	23, 587

### 3.2 Data pre-processing

Among the issues with tweet data is the noise that is streamed along with the tweets. Tweets come in diverse forms, encompassing text, user mentions, URLs, and hashtags (#). At this phase, the HS and non-HS data from both the Arabic dataset and the code-mixing dataset underwent pre-processing to prepare the data for the subsequent feature extraction and classification stages. Pre-processing is an essential stage when handling noisy and informal data, such as Twitter content. This is particularly critical when dealing with Arabic, which is inherently ambiguous and features a broad range of dialects used in the Twittersphere. The pre-processing stage involved several steps aimed at eliminating noise. The textual data underwent transformations like converting to lowercase, along with other fundamental pre-processing methods like tokenization, elimination of stop words, and spell checking. The preprocessing techniques conducted are described below.

- Data cleaning: In this step, punctuation, extra whitespace, user mentions, repeated characters, emojis, and diacritics are removed.
- Hashtag removal: This measure was implemented to eliminate potential bias toward specific classes; we excluded any hashtags utilized as keyword searches during the collection of tweet data.
- Remove stop words: These are the frequently occurring words that occur excessively and typically do not offer meaningful information in text classification, including prepositions, articles, and conjunctions. In this study, we utilized the Arabic stop word list from NLTK to eliminate stop words. Multiple research studies have shown that the inclusion of stop words negatively impacts modeling effectiveness. Consequently, the removal of stop words was implemented to enhance the modeling outcomes. The feature extraction stage is next to the pre-processing step.
- Normalization: The primary goal of this method is to generate more uniform tweets. In the Arabic language, there are various variations to portray certain letters, including
  - to. Letter (Alef) (أ) which possesses the forms (أ-إ-آ-ا) we standardize all these four letters to a single letter, which is (ا).
  - Letter (Alef Maqsora) (ى) that can be confused and written as (ي). The normalization form of it will be (ي).
  - The letter (Taa Marbouta) (ة) normalization form will be (ه). In the end, there will be the inclusion of the removal of the Arabic script.

### 3.3 Data annotation

**Fig 3:**
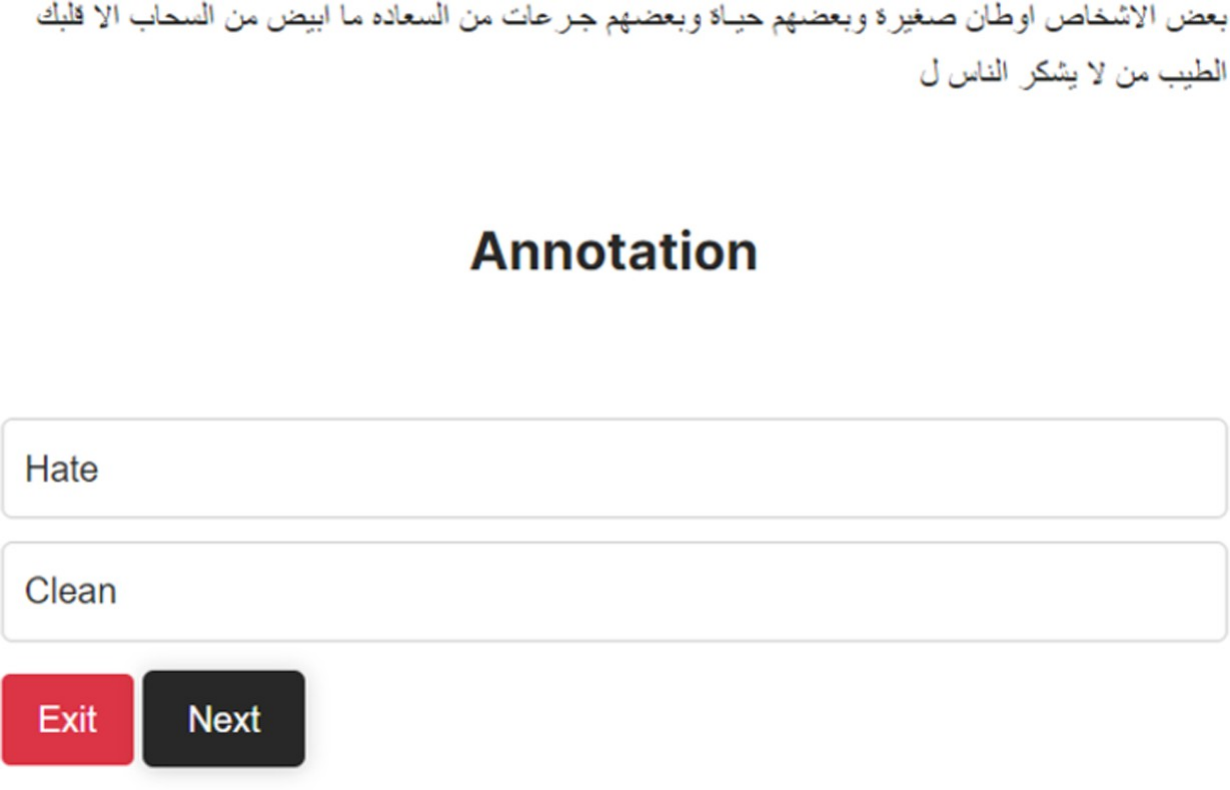
Web portal for manual data labeling.

Upon the completion of data collection, the next step was to prepare the data for annotation. The dataset was divided into two categories: Arabic and code-mixed tweets (tweets that have code-mixed words). To label the data, a hybrid approach that combines manual and automatic labeling was employed. To facilitate manual labeling, an online portal was created with the aid of Python, as shown in Fig 3. After uploading 20% of the data to the system, each tweet was displayed on the page, allowing users to label each sentence as either hateful or not. We selected eight individuals who were fluent in Arabic from two groups, each consisting of four students, to annotate the data. Prior to presenting the task to the annotators, we worked together with experts in interreligious discourse, Islamic jurisprudence, and media studies to create an annotation guideline tailored for HS. During the processing period, the Kappa coefficient was utilized to measure the inter-agreement between them [36]. During the processing period, the Kappa coefficient was utilized to measure the inter-agreement between them [37].

### 3.4 Handling code-mixing

When dealing with code-mixed words, one effective method is to employ transliteration based on a dictionary-based approach. This technique involves utilizing existing resources and techniques. Although there is limited research on transliteration-based methods for Arabic-English code-mixed data, the employment of such mechanisms remains a significant hurdle to overcome when processing and analyzing code-mixed social media text [38]. Dictionary-based transliteration methods are widely used and compared with other techniques like rule-based and web-based n-best re-ranking. Research shows that the pronunciation dictionary used in transliteration methods has a significant impact on accuracy [39].

**Fig 4:**
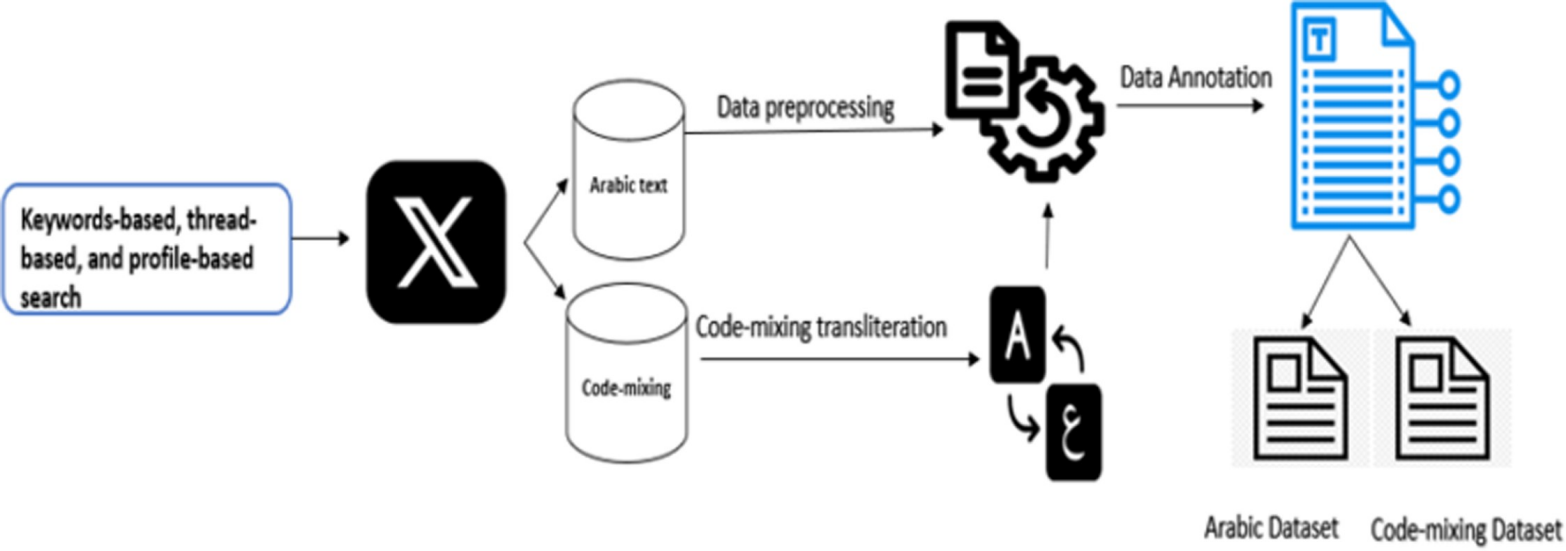
Arabic hate speech corpus creation.

Therefore, to effectively tackle code mixing in our dataset, we implemented a careful two-step approach. The first step is to carefully create a comprehensive dictionary tailored specifically to code-mixed words. Creating a code-mixing dictionary containing 554 words in the Saudi dialect is an impressive accomplishment that demands a profound comprehension of both Arabic and English, along with the intricacies of code-mixing. This dictionary would cover a broad spectrum of vocabulary, including common terms and phrases used in daily conversations, as well as slang and colloquialisms specific to the Saudi dialect. It is important to note that code-mixing is a complex linguistic phenomenon when two or more languages are mixed inside a single utterance or document [40]. The dictionary contains an explicit mapping of the representations of code-mixed words to their corresponding Arabic transliterations. The second stage involves developing and using a Python script to transliterate the mixed code words based on the established dictionary. The script integrates seamlessly into the data preprocessing pipeline and systematically replaces identified code-mixing terms with accurate Arabic transliterations. The combination of fine-tuned dictionaries and purpose-built Python scripts not only ensures accuracy in handling instances of code-mixing but also enables an efficient and scalable transliteration process within the broader context of HS detection in code-mixing datasets. Fig 4 depicts the procedures we followed to create the two datasets.

### 3.5. Feature extraction

The classification criteria in the raw text are not comprehensible to the machine learning models. For these classification models to comprehend classification rules, a numerical feature is required. Thus, feature extraction is the most important phase in a text mining study. The main features of the raw text are retrieved using this process, and the extracted features are then represented numerically. At the feature engineering phase, the data that was processed is examined for features with the ability to discriminatively distinguish hate speech from non-hate speech. This phase also investigates various feature engineering approaches, including feature representation and subset feature selection, in addition to feature extraction [41]. Prior research has focused on content-oriented features, such as Bag-of-Words features, without taking contextual factors into account when detecting HS. According to analysis results using content features, not all of the HS tendencies in the text can be accurately captured by these attributes only. To improve the model’s performance, a few extensive features have been suggested to supplement the content features. Three distinct feature engineering strategies have been used in this study: N-gram, TF-IDF, and CBOW. The features were derived from the content of the tweets. These features, outlined below, were utilized in combination with the machine learning classifiers to build a model for identifying HS. A detailed description of each of the features is provided in the subsection below.

#### 3.5.1 N-gram feature

In order to extract N-gram features, a text must be divided into contiguous word sequences of N in order to identify local semantic correlations and patterns [42]. Tokenizing the text, changing its case, and eliminating unwanted characters are the first steps in this procedure. Next, N-grams of the desired size are produced by overlaying a tokenized text window with N words, forming sequences of related word combinations. N-grams are the sequences that are utilized as the extracted features. The sentence "natural language processing" for bigrams (N = 2), for example, would result in the bigrams "natural language" and "language processing." This feature extraction technique is useful for identifying relationships and context between words in a document [43,44].

#### 3.5.2 TF-IDF feature

With the use of the TF-IDF technique, significant features can be extracted from the gathering of documents by measuring the relevance of individual words. There are multiple essential steps in the process of retrieving TF-IDF features. Preprocessing is first applied to the raw text data, which includes tokenization, converting to lowercase, and eliminating special characters [45]. Then, to enhance the relevance of the extracted features, stop words (words that are frequently used but not very informative) are removed. The Term Frequency and Inverse Document Frequency components are then combined to determine the TF-IDF value for each term. A term’s frequency of occurrence in a document is measured by TF, but its significance throughout the entire document collection is evaluated by IDF. The relevance of every term in a particular document has a number represented by the TF-IDF score, which is calculated by multiplying TF and IDF. Following the computation of TF-IDF values for each term in each document, the data is frequently shown in a matrix style, with every row representing a document and each column indicating a unique term [46]. This matrix functions as the text data’s TF-IDF feature representation, encapsulating the unique qualities of every document according to the significance of its terms. Because they give a quantitative measure of word relevance in a particular context, the resulting TF-IDF characteristics serve as a foundation for a variety of NLP tasks, such as text mining, information retrieval, and opinion mining.

#### 3.5.3 CBOW feature

CBOW feature: CBOW is one of the architectures of word2vec [47]. CBOW is a “neural network” model. It considers the words in the context around a target word to predict that specific word. It is a shallow neural network where the input is the context words, and the output is the target word [48]. CBOW features are typically extracted either through the use of a pre-trained model or by training a model on a substantial corpus of text. Tokenization, preprocessing, lowercase word conversion, and character removal from superfluous text are all part of the process. Following that, the CBOW model is built, with parameters like vector size and window size defining the word embeddings’ and the context window’s respective dimensions [49,50]. Based on the context around a target word, the training of the model is done by the pre-processed text data. By acquiring the dense vectors that each word in the vocabulary represents after it has been trained, CBOW features can be extracted. Utilizing contextual information and semantic linkages for every word, these dense vectors function as the CBOW features. CBOW is an enhanced edition of the BOW technique. The difference between the BOW and CBOW is that BoW is a simple and order-agnostic method for representing text, whereas CBOW is a neural network-based model that considers word order and context to create word embeddings. The CBOW model is swifter and proficient in representing frequently occurring words [51].

### 3.6. Machine learning models

This section provides a description of the classification models utilized in this research. In this study, the binary approach classifies data into two possible classes. In order to achieve a binary classification, feature extraction from the data is required in order to construct the ML model for HS classification. The construct ML model takes the features as an input and outputs the class that the feature belongs to. As per the "no free lunch theorem" [52], there exists no one ML algorithm that exhibits optimal results across various datasets. As a result, it is advised to utilize as many classifiers as possible. To find the best classifier for HS identification, a variety of classification algorithms, including LR, NB, RF, KNN, and SGD, with a majority voting ensemble, were examined in various experiments. It can be difficult to decide which classifier is appropriate for a given dataset, though. Because it is challenging to identify a single classifier that can achieve the highest accuracy in every application domain, two or more ML models are examined in the current studies to determine which model performs better [53]. This is because of differences in the learning process’s pedagogy. Therefore, to ascertain the algorithm’s effectiveness of the Arabic HS detection model, five distinct models have been used, including LR, NB, RF, KNN, and SGD. These algorithms are described below.

**Logistic regression** (LR): Logistic regression is a predictive algorithm that linearly predicts the likelihood of an event by considering a set of predictor variables [41]. An attribute-based linear function is typically used in the LR method to determine the decision bounds. The goal of logistic regression is to improve the likelihood function for identifying the document class label. Attaining the maximum conditional probability is the aim of selecting parameters in the LR [54]. Although LR yields a promising result, the class variable that is typically created falls outside of the probability limit (0–1).

**K-nearest neighbor (KNN):** Typically used for regression and classification problems, the KNN algorithm is an instance-based predictive model. The KNN of each instance determines the instance’s class label in this type of modeling. Therefore, the neighbor instance uses the ‘majority voting’ method in order to ascertain the class label. Nevertheless, the class instance of each neighbor—that is, the most common class instance of the k-nearest neighbor—is given the majority vote in this categorization scheme [55].

**Random Forest (RF):** RF is an ensemble classifier that has drawn more attention because of its accuracy and resistance to noise when it is compared to a single classifier. The RF works by combining several decision trees into one powerful ensemble classifier. The random forest gains unique characteristics from the idea of merging numerous classifiers, which significantly sets it apart from conventional tree classifiers. Because of its resilience to noise and outliers, the RF classifier offers randomness to mitigate issues similar to those encountered by a single DT algorithm with outliers, which could potentially impact the predictive accuracy of the model. In addition to adding unpredictability to the input, the random forest also adds features. Similar ideas from bootstrapping and bagging classifiers are used in random forests. This is accomplished by making trees more diverse, which leads to their growth from various training data subsets produced by bootstrap aggregation [56].

**Naïve Bayes (NB):** The NB Model makes the assumption that the characteristics of data values are both strongly and weakly independent. Its foundation is the Bayes theorem, which states that given evidence, a hypothesis has a given probability. When it comes to text classification, the words in the document serve as the evidence, and the class label serves as the hypothesis. Because NB believes that features (words) do not depend on the class label based on the condition and as a result, the analysis is deemed tractable. Since it is a classifier based on probability, its predictions are made in accordance with the likelihood that an object will occur [44]. Despite the fact that the assumption of conditional independence is rarely valid, one advantage of NB models is that they perform well [57].

### Stochastics Gradient Decent

Stochastic Gradient Descent (SGD), for short, is an optimization technique that is frequently used for model training in deep learning and machine learning. SGD is an optimization technique that varies on the gradient descent process [58]. Rather than calculating the gradient of the complete dataset, it does it using a randomly chosen portion of the data, known as a mini-batch. SGD’s fundamental principle is to adjust the model’s parameters in a way that minimizes the cost function with regard to the chosen subset of data. With large datasets in particular, this stochastic technique can assist in breaking out of local minima and accelerate convergence by introducing unpredictability into the optimization process.

## 4. Experimental setup

The purpose of the classification experiment was to categorize tweets into hate and non-hate categories. Every experiment was carried out using a MacBook Pro machine. Using an Apple M1 chip with an 8-core CPU (unique ARM-based architecture), an 8-core GPU, and 8GB of RAM, the system operates. Python development platforms such as Jupyter Notebook were used to carry out the pre-processing and feature extraction operations. For various ML models, different subsets of attributes were used as inputs. in the HS analysis experiment. The study involved the application of five distinct machine learning models, namely LR, RF, KNN, SGD, and NB, to ascertain the presence of HS in the provided tweets. The goal behind utilizing various algorithms is to achieve optimal predictive outcomes. We applied an 80 by 20 split to divide the clean data and allocate it for training and testing data. Test data is used to evaluate the accuracy of the learning algorithm, while training data is used to train the model on new categorization rules. Anaconda was the platform used for the analysis and feature extraction studies, and the experiment was conducted with the default settings kept constant. To evaluate the models’ performance, five standard metrics, namely AUC, precision, recall, f-measure, and accuracy, were employed for evaluation and provided appropriate weighting.

As outlined in subsection 3.5, this study utilized three sets of variant features, namely n-gram (unigram, bigram, and trigram), TFIDF, and CBOW, resulting in three distinct master feature representations. Furthermore, we applied five diverse machine learning algorithms to each of the three master feature vectors for both the Arabic dataset and the code-mixing dataset. Consequently, a total of 30 analyses (comprising 3 master feature vectors multiplied by 5 machine learning algorithms) were conducted for each dataset to assess how effective the machine learning algorithm can detect HS.

### 4.1 Evaluation metrics

Evaluation metrics are performance indicators for measuring analysis outcomes. A range of matrices were applied to evaluate the classifiers’ performance. In this study, the key performance evaluation criterion is ’accuracy.’ However, additional performance metrics, including AUC, recall, precision, and f1-score, were utilized as supplementary measures to assess the effectiveness of the proposed method. Each learning model demonstrates its power in identifying HS when evaluated using these metrics. In this phase, the classification models make a class prediction on the unlabeled text (i.e., whether it falls into the categories of "hate speech" or "not hate speech") using the testing set of data. The models’ effectiveness is measured by computing the counts of true negatives (TN), false positives (FP), false negatives (FN), and true positives (TP). These terms collectively form a confusion matrix, illustrated in Table 3. Various evaluation metrics are employed to evaluate the effectiveness of the classification models. Brief descriptions of these matrices are provided below. Additional details on these performance metrics can be found in [8].

**Table 3:** Confusion matrix.

	Predicted Not Hate	Predicted Hate
Actual Not Hate	True Negative (TN)	False Positive (FP)
Actual Hate	False Negative (FN)	True Positive (TP)

Accuracy: Accuracy serves as a metric to gauge a classifier’s ability to accurately predict instances of Hate and Not Hate within each class. It is determined by the ratio of correctly classified instances to the overall instances, as expressed in Eq 1.


$$\text{Accuracy}=\frac{\text{TP}+\text{TN}}{\text{TP}+\text{FP}+\text{TN}+\text{FN}}$$ (1)

Precision: Precision is the proportion of instances that were accurately predicted to the overall predicted instances and is computed as shown in Eq 2.


$$\text{Precision}=\frac{\text{TP}}{\text{TP}+\text{FP}}$$ (2)

Recall: It represents the proportion of instances (Hate and Not Hate) classified that were accurately predicted out of the total number of actual instances. The calculation is performed following the formula given in Eq 3.


$$\text{Recall}=\frac{\text{TP}}{\text{TP}+\text{FN}}$$ (3)

F1-score: The F1-score integrates Precision and Recall, providing a balanced average of these two metrics and serving as an aggregated performance score. The calculation of the F1-sore for a class C, where C represents either Not Hate or Hate, is determined following the formula given in Eq 4.


$$\text{F}1-\text{score}=2*\left(\frac{\text{Precision}*\text{Recall}}{\text{Precision}+\text{Recall}}\right)$$ (4)

AUC-ROC Curve—Referred to as ROC, the Receiver Operator Characteristic is a graphical representation of the true positive rate (TPR) against the false positive rate (FPR) across various threshold values. The area under the ROC curve (AUC) serves as a metric to quantify the points on this curve, indicating the capacity of the classifier to differentiate between classes. In our binary classification study, this metric is particularly valuable for identifying the most effective classifier. Eqs (5) and (6) outline the formulas used to compute the TPR and FPR.


$$\text{TPR}=\frac{\text{TP}}{\text{TP}+\text{FN}}$$ (5)

$$\text{FPR}=\frac{\text{FP}}{\text{FP}+\text{TN}}$$ (6)

## 5. Results and discussion

The predictions made by the 30 analytic experiments on datasets are presented and discussed in this section. Initially, we looked at two datasets: HS in Arabic and HS that involved code-mixing. N-gram, TF-IDF, and CBOW are a few of the feature engineering techniques that were initially used to appropriately preprocess these datasets in order to modify the data in an effective manner. A comparison study was presented and reviewed by evaluating each of the five machine learning models using the suggested approach and models, as well as the f1-score, accuracy, recall, precision, and ROC curves. The accuracy, precision, recall, f1-score, and AUC of each of the 30 analyses are displayed in Tables 4 through 7 accordingly. The majority of the experiment’s findings were excellent and helped determine which classifiers would perform the best for HS classification. The highest and lowest possible outcome values are indicated by the bold values. The results for various feature representations and classifiers used in experimentation scenarios are displayed in each table.

**Table 4:** Performance results comparison obtained on N-gram feature on Arabic and code-mixing.

	Arabic Tweets dataset					Code-Mixing Tweets Dataset				
Models	ACC	PRE	REC	F1-SC	AUC	ACC	PRE	REC	F1-SC	AUC
**LR**	0.9199	0.9199	0.9199	0.9200	0.9754	0.9511	0.9519	0.9511	0.9508	0.9839
**RF**	0.8845	0.8845	0.8845	0.8811	0.9309	0.8897	0.8962	0.8897	0.8903	0.9467
**NB**	0.8244	0.8244	0.8244	0.8289	**0.9880**	0.9219	0.9326	0.9219	0.9225	0.9949
**SGD**	**0.9350**	**0.9350**	**0.9350**	**0.9341**	0.9820	**0.9685**	**0.9687**	**0.9685**	**0.9684**	**0.9932**
**KNN**	0.6515	0.6515	0.6515	0.5849	0.7500	0.7940	0.8530	0.7940	0.7814	0.7753

### 5.1 Results

#### 5.1.1 Results based on N-gram features

**Fig 5:**
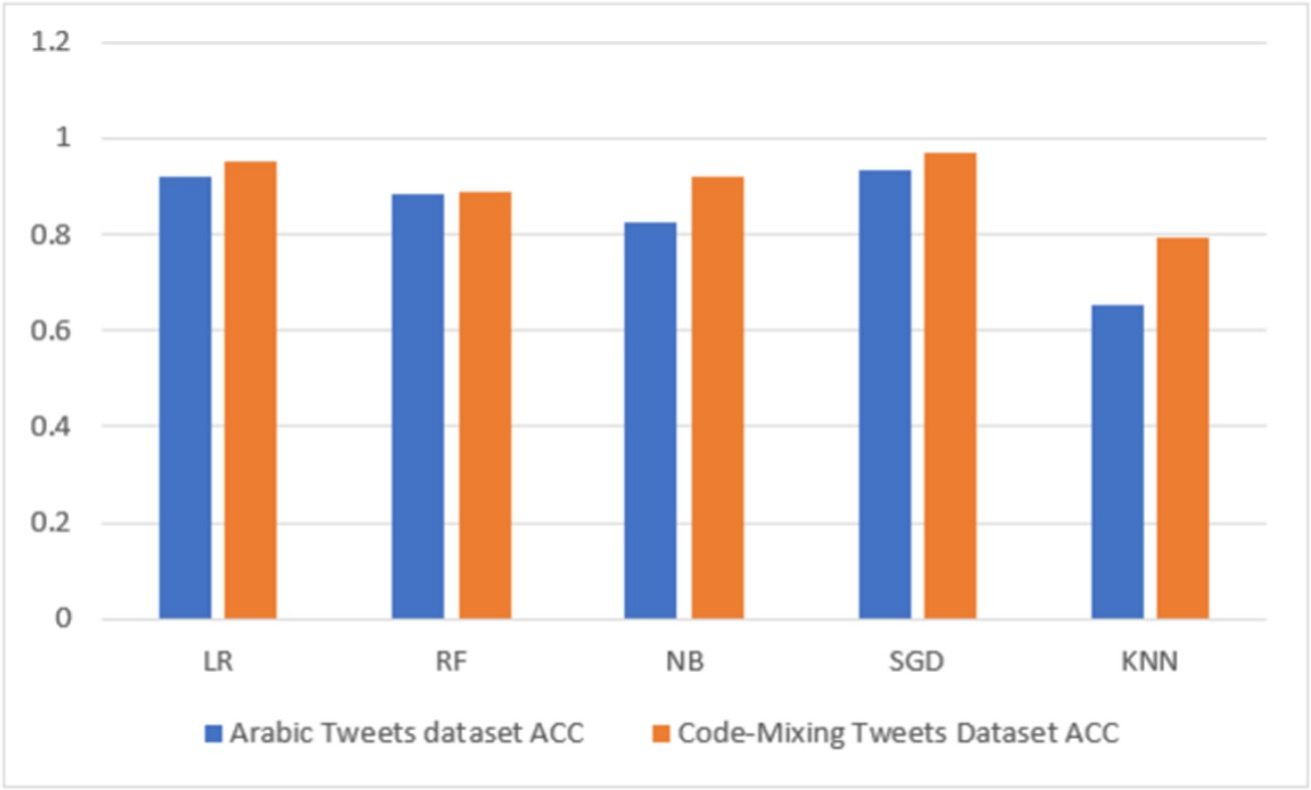
N-gram highest accuracy result visualization.

The classification performance obtained on N-gram is shown in Table 4, and the visual representation of the results is shown in Fig 5. As shown in Table 4, the proposed method was effective in the model evaluation. The table shows the predictive result by comparing different classifiers’ performance on the Arabic HS dataset and the code-mixing dataset for HS detection. In all 10 analyses conducted, it can be found that the lowest accuracy (0.6515), precision (0.6515), recall (0.6515), F1-score (57%), and AUC (0.47) are found in the KNN classifier on the Arabic HS dataset, whereas in the code-mixing HS dataset, the lowest performance accuracy (0.7940), precision (0.8530), recall (0.7940), f1-score (0.7814), and AUC (0.7753) are also found in the KNN classifier. On the other hand, it can also be observed from Table 4 that the highest performing accuracy (**0.9350**), precision (**0.9350**), recall (**0.9350**), f1-score (**0.9341**), and AUC (**0.9880**) are found in the SGD and NB classifiers on the Arabic HS dataset, whereas in the code-mixing HS, the highest performing accuracy (0.9685), precision (0.9687), recall (0.9685), f1-score (0.9684), and AUC (0.9949) are also found in the SGD and NB classifiers. It can be seen in the summary results for the N-gram feature experiment, as shown in Table 4 for both datasets, that the code-mixing dataset outperformed the Arabic dataset for all the classification models experimented with. We can see the significance of the code-mixing by enhancing the percentage accuracy by 3.35%, precision by 3.37%, recall by 3.35%, f1-score by 3.43%, and AUC by 0.69%, respectively.

#### 5.1.2 Results based on TF-IDF features

**Fig 6:**
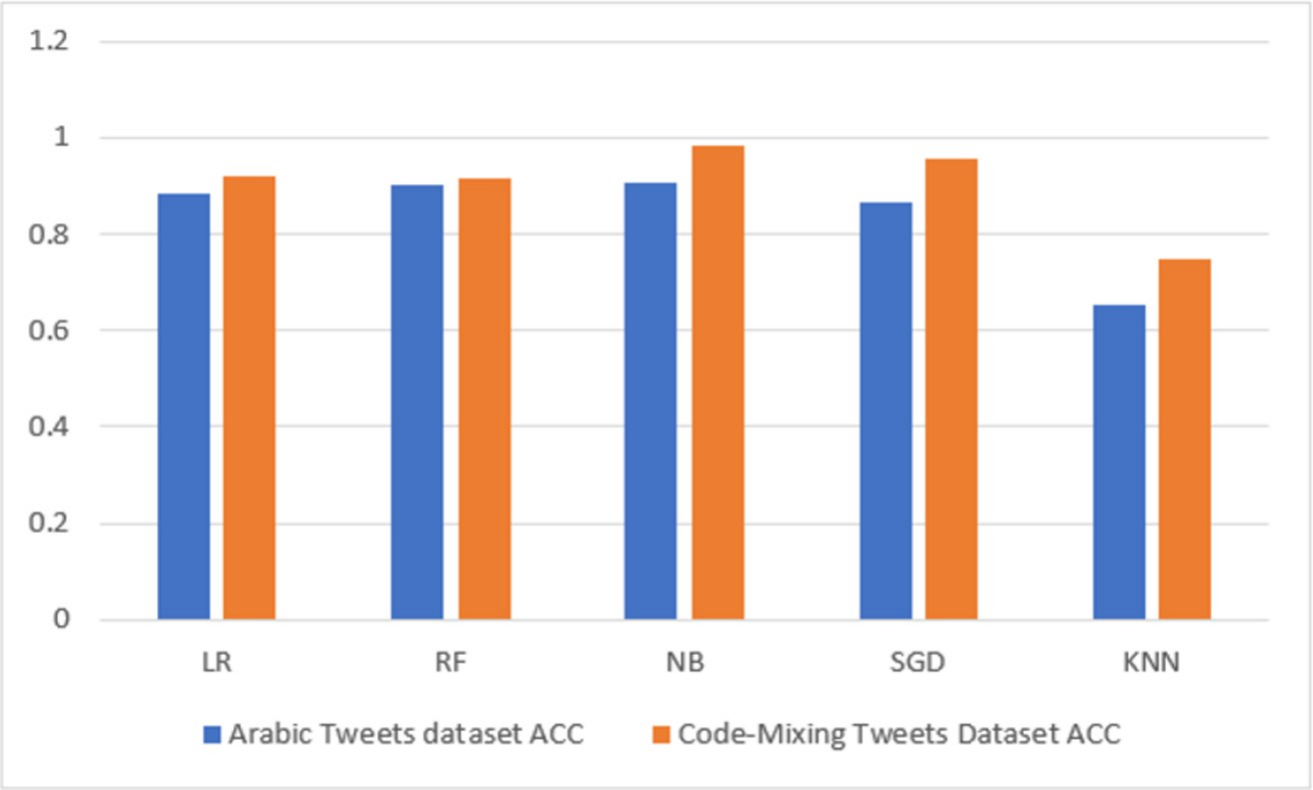
TF-IDF highest accuracy result visualization.

Table 5 and Fig 6 also present the classification results obtained based on the TF-IDF features. As shown in Table 5, the proposed method was successful in evaluating the model. The table compares several classifiers and displays the predicted outcome on the Arabic HS dataset and the code-mixing dataset for HS detection. In all the 10 analyses conducted, it can also be observed from Table 5 that the highest performing accuracy (0.9061), precision (0.9061), recall (0.9061), f1-score (0.9077), and AUC (0.9818) are found in the NB classifier on the Arabic HS dataset, whereas in the code-mixing HS, the highest performing accuracy (0.9821), precision (0.9823), recall (0.9821), f1-score (0.9820), and AUC (0.9940) are also found in the NB classifier. On the other hand, it can be found that the lowest accuracy (0.6515) precision (0.6515), recall (0.6515), F1-score (5849), and AUC (0.7373) are found in the KNN classifier on the Arabic HS dataset, whereas in the code-mixing HS dataset, the lowest performance accuracy (0.7505), precision (0.7970), recall (0.7505), f1-score (0.7344), and AUC (0.7296) are also found in the KNN classifier. It can be seen in the summary results for the TF-IDF feature experiment, as shown in Table 5, that the code-mixing dataset outperformed the Arabic dataset for all the classification models experimented with. Thus, we can see the significance of the code-mixing in enhancing the percentage accuracy by 7.6%, precision by 7.63%, recall by 7.6%, f1-score by 7.43%, and AUC by 1.22%, respectively.

**Table 5:** Performance results comparison obtained on TF-IDF feature for Arabic and code-mixing.

	Arabic Tweets dataset					Code-Mixing Tweets Dataset				
Models	ACC	PRE	REC	F1-SC	AUC	ACC	PRE	REC	F1-SC	AUC
**LR**	0.8856	0.8856	0.8856	0.8868	0.9462	0.9212	0.9216	0.9212	0.9214	0.9712
**RF**	0.9028	0.9028	0.9028	0.9006	0.9349	0.9166	0.9204	0.9166	0.9171	0.9579
**NB**	**0.9061**	**0.9061**	**0.9061**	**0.9077**	**0.9818**	**0.9821**	**0.9823**	**0.9821**	**0.9820**	**0.9940**
**SGD**	0.8669	0.8669	0.8669	0.8685	0.9464	0.9576	0.9576	0.9576	0.9575	0.9904
**KNN**	0.6515	0.6515	0.6515	0.5849	0.7373	0.7505	0.7970	0.7505	0.7344	0.7296

#### 5.1.3 Results based on Word Embedding (CBOW) features

**Fig 7:**
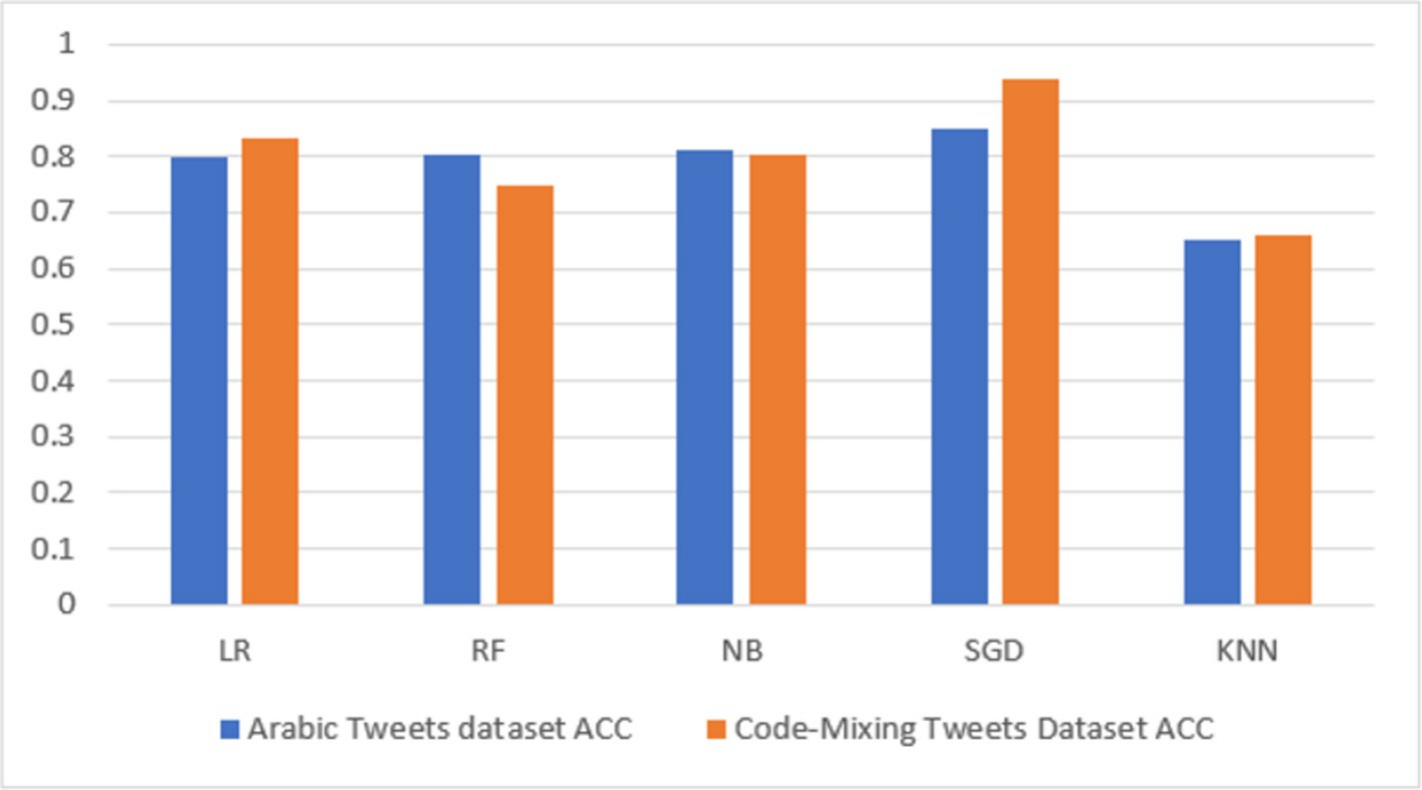
CBOW highest accuracy result visualization.

Table 6 and Fig 7 also present the model performance obtained based on the CBOW embedding features. As shown in Table 6, the proposed method was successful in evaluating the model. The table compares several classifiers and displays the predicted outcome on the Arabic HS dataset and the code-mixing dataset for HS detection. In all the 10 analyses conducted, it can also be observed from Table 6 that the highest performing accuracy (0.8482), precision (0.8482), recall (0.8482), f1-score (0.8505), and AUC (0.9244) are found in the SGD classifier on the Arabic HS dataset, whereas in the code-mixing HS, the highest performing accuracy (0.9382), precision (0.9382), recall (0.9382), f1-score (0.9382), and AUC (0.9793) are also found in the SGD classifier. On the other hand, it can be found that the lowest accuracy (0.6515) precision (0.6515), recall (0.6515), F1-score (5849), and AUC (0.6313) are found in the KNN classifier on the Arabic HS dataset, whereas in the code-mixing HS dataset, the lowest performance accuracy (0.6615), precision (0.5960), recall (0.6615), f1-score (0.5772), and AUC (0.6136) are also found in the KNN classifier. It can be seen in the summary results for the CBOW feature experiment, as shown in Table 6, that the code-mixing dataset outperformed the Arabic dataset for all the classification models experimented with. Thus, we can see the significance of the code-mixing in enhancing the percentage accuracy by 9%, precision by 9%, recall by 9%, f1-score by 8.77%, and AUC by 5.51%, respectively.

**Table 6:** Performance results comparison obtained on CBOW feature for Arabic and code-mixing.

	Arabic Tweets dataset					Code-Mixing Tweets Dataset				
Models	ACC	PRE	REC	F1-SC	AUC	ACC	PRE	REC	F1-SC	AUC
**LR**	0.7971	0.7971	0.7971	0.8015	0.8828	0.8344	0.8346	0.8344	0.8345	0.9163
**RF**	0.8037	0.8037	0.8037	0.7898	0.8401	0.7470	0.7905	0.7470	0.7477	0.8337
**NB**	0.8131	0.8131	0.8131	0.8167	0.8931	0.8011	0.8029	0.8011	0.8016	0.8873
**SGD**	**0.8482**	**0.8482**	**0.8482**	**0.8505**	**0.9244**	**0.9382**	**0.9382**	**0.9382**	**0.9382**	**0.9795**
**KNN**	0.6515	0.6515	0.6515	0.5849	0.6313	0.6615	0.5960	0.6615	0.5772	0.6136

### 5.2 Discussions

In this classification experiment study, we have evaluated five classification algorithms on three variant features, by providing 30 various analyses on Arabic HS and code-mixing datasets with two classes (hate and not hate). The results of the experiment indicated that the SGD algorithm with TF-IDF feature engineering technique attained the best result on the code-mixing dataset by producing 98.18% accuracy. The sub-section below provides a discussion of the theoretical analysis of the results based on feature engineering and machine learning classifiers.

#### 5.2.1 Feature engineering

In text mining studies, the choice of feature engineering is crucial. In this research, three different feature engineering approaches were compared, including the N-gram (unigram, bigram, and trigram) feature, the TF-IDF feature, and the word embedding (CBOW) feature. The results of the experiment exhibited that out of the three feature engineering techniques tested in the experiment, the TF-IDF feature engineering techniques outperformed the N-gram and CBOW features for both the Arabic HS and code-mixing datasets based on the evaluation metrics used in this study. The reason behind the exceptional performance of the TF-IDF in detecting HS in Arabic tweets is due to its sensitivity to the particular linguistic features of Arabic [59]. Because Arabic is a highly contextual and morphologically rich language, a word’s meaning might change depending on where it appears in a sentence and its context. The peculiarities of HS in Arabic are especially well captured by TF-IDF because of its capacity to account for term frequency inside individual tweets and their inverse frequency throughout the entire dataset. By identifying a term’s importance within the context of individual tweets, this feature aids in the classifier’s ability to identify subtle linguistic details linked to HS in the Arabic language.

Moreover, the second highest performing feature is the N-gram feature, which consists of unigram, bigram, and trigram. The possible reason for the outperformance of the N-gram over the CBOW feature is that the N-gram, especially the bigram, retains the word sequence compared to CBOW. Conversely, the CBOW feature indicated lower results. The possibility is that the CBOW may not be as effective in detecting HS in Arabic because of issues with the morphological complexity of the Arabic language and the small context that CBOW takes into account [36]. Arabic has complex morphological variants, meaning that a word’s form can vary greatly depending on its context and grammatical characteristics. It may be difficult for CBOW, which predicts a target word based on its context, to pick up on the many subtle variations of Arabic words.

#### 5.2.2. Machine learning classifier

It was demonstrated by numerous experiments that no single machine learning method outperformed the others when applied to diverse types of data. As a result, comparing different machine learning models is necessary to determine which one works best with the particular dataset. Thus, as covered in Section 3.6 (Machine Learning Models), we applied five distinct machine learning models to our dataset. The experimental findings demonstrated that SGD and NB classifiers performed the best. Out of the five models tested, the SGD model shows noteworthy effectiveness in HS detection in Arabic. The possible reason could be due to its flexibility in handling high-dimensional and sparse feature spaces [60], which is one of the common issues in natural language processing tasks. Consequently, the iterative optimization of the SGD model helps it to efficiently navigate the complicated feature environment and settle on optimal parameter values in Arabic HS detection, where linguistic subtleties and contextual nuances are important. In addition, the SGD works well with big datasets, which is helpful for the Arabic datasets, which are frequently enormous and varied. Therefore, the SGD model is a good option for Arabic HS identification applications because of its computational efficiency and capacity to handle noisy and unstructured data. The model’s impressive performance on these datasets can be attributed to its adaptability in assimilating the various linguistic patterns found in Arabic HS. On the other hand, Naive Bayes is a good choice for text classification jobs since it works under the premise that features should be independent of the class label [61]. When it comes to Arabic HS identification, Naive Bayes effectively captures the probabilistic correlations between phrases and their recurrence in situations that either support or oppose HS. This is because Arabic language patterns can be quite complex. Conversely, the KNN performed the poorest [62] among all the classification models because of the laziness of the classifier and its inability to function well with noisy input. Thus, it is not appropriate to use the KNN to identify HS in tweets.

#### 5.2.3 Comparison of the proposed approach with the baselines

**Fig 8:**
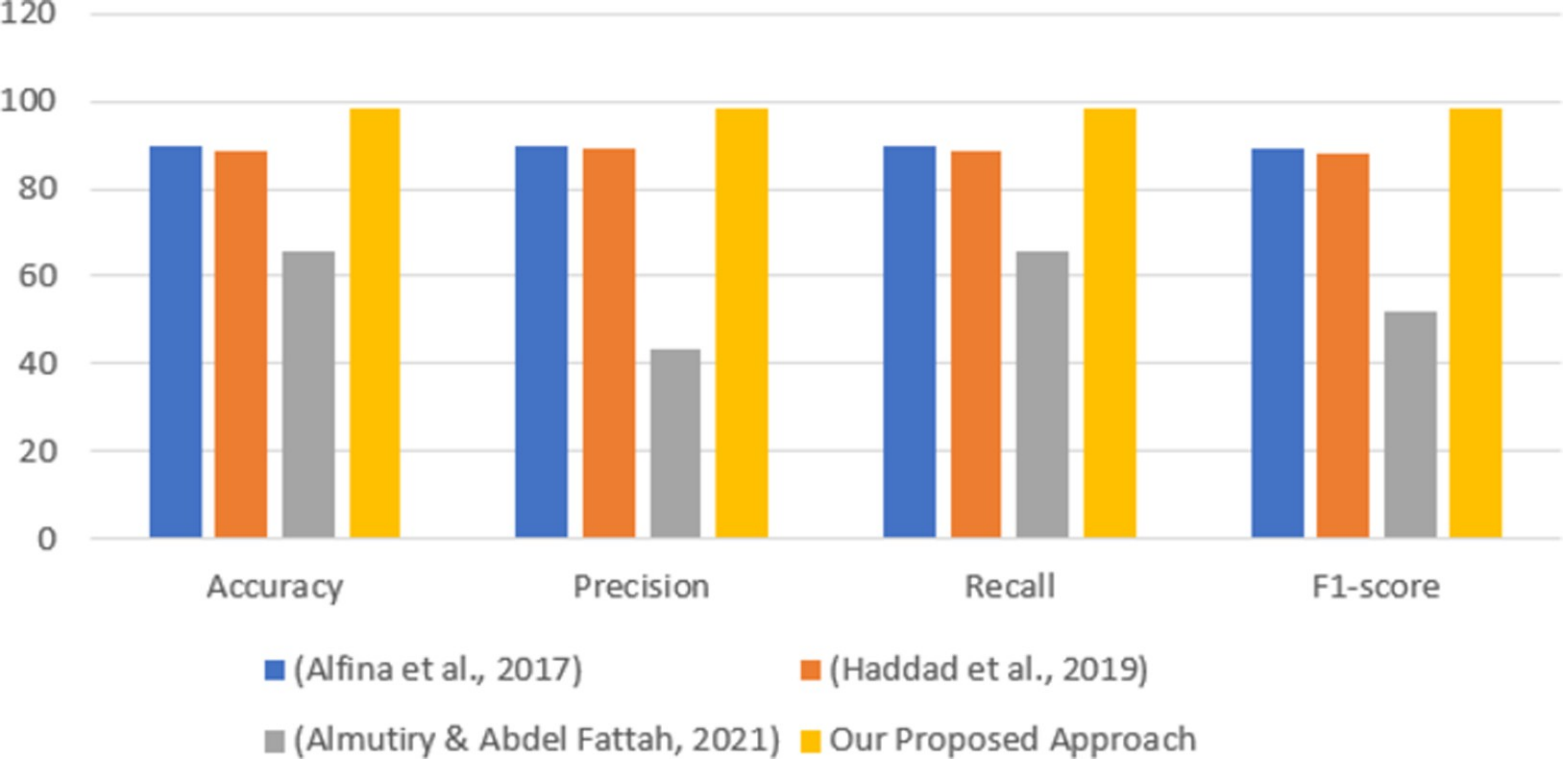
Comparison of the proposed method with baselines.

To assess the importance of the proposed methodology, a comprehensive set of experiments was conducted on the dataset utilized in this research to assess the performance of ML models employing three established approaches for identifying HS in Twitter data. Three baseline approaches were implemented for comparison with the proposed method. The initial baseline approach is derived from the research conducted by [63], incorporating a combination of N-gram and Bag-of-Words (BoW) features. The second baseline draws inspiration from the study by [64], which integrates N-gram and Term Frequency-Inverse Document Frequency (TF-IDF) features. Meanwhile, the third baseline relies solely on the TF-IDF feature, as outlined in [65]. In the comparison experiments, the initial baseline demonstrated outstanding performance, achieving an accuracy of 89.62% with an RF classifier. In the second baseline, a random forest classifier yielded the optimal performance, achieving an accuracy of 88.38%. The third baseline secured the lowest outcome with an SVM classifier, recording an accuracy of 65.64%. We contrasted the optimal results attained from our proposed approach with those achieved by three baseline studies. The comparative performance is illustrated in Table 7, with the last row of the table showcasing the results of our proposed approach. Using the SGD classifier, our proposed method achieved the highest accuracy of 98.21% with the TF-IDF feature, underscoring the importance of our method for HS identification. Consequently, our proposed method surpassed the first baseline by 8.65%, the second baseline by 9.85%, and the third baseline by 32.57% in terms of accuracy. Moreover, our method also outperformed the baselines in terms of precision, f1-score, and recall when contrasted with the baselines. The visual representation of this comparison is depicted in Fig 8.

**Table 7:** Comparison of the proposed approach with the baselines.

Studies	Accuracy	Precision	Recall	F1-score
[63] bow+n-gram	89.56	89.62	89.56	89.35
[64] N-gram +TF-IDF	88.38	88.96	88.38	87.93
[65] TF-IDF	65.64	43.09	65.64	52.03
Our Proposed Approach	**98.21**	**98.23**	**98.21**	**98.20**

## 6. Conclusion

Hate speech (HS) identification is one of the major challenges in natural language processing research. Even with the strong measures taken to prohibit HS on social media, HS is still a problem that needs to be acknowledged and closely examined. The Arabic language presents distinct obstacles due to its various dialects and grammatical peculiarities, which emphasizes the need for specific methodologies in the identification of HS. The necessity for sophisticated solutions is further highlighted by the extra layer of complexity brought about by the common practice of code-mixing. Filling in this research gap, this research utilized automatic text classification methods for HS identification in Arabic tweets. In addition, this research compares the effectiveness of different features, including TF-IDF, N-gram, and CBOW, and machine learning models, consisting of NB, RF, SGD, KNN, and LR, for HS detection on Arabic HS and code-mixing HS datasets. The findings from the analysis revealed that the TF-IDF feature, when employed with the SGD model, attained the highest accuracy, reaching 98.21%. Subsequently, these results were contrasted with outcomes from three existing studies, and the proposed method outperformed them, underscoring the significance of the proposed method. Furthermore, this study carries practical implications and serves as a foundational exploration in the realm of automated HS detection in text. The limitation of the proposed method is that even though it can detect HS, it cannot detect the severity of the HS expression. Hence, in future studies, the author will improve the proposed method, which can be utilized to detect the severity of the HS expression. Furthermore, we will apply a multi-label classification approach for HS in code-mixing tweets.

## Supporting information

S1 Table (PDF)
