# Correction: Code-mixing unveiled: Enhancing the hate speech detection in Arabic dialect tweets using machine learning models

**DOI:** 10.1371/journal.pone.0330305

**Published:** 2025-08-13

**Authors:** Ali Alhazmi, Rohana Mahmud, Norisma Idris, Mohamed Elhag Mohamed Abo, Christopher Ifeanyi Eke

The Funding statement for this article is incorrect. The correct Funding statement is as follows: The authors gratefully acknowledge the funding of the Deanship of Graduate Studies and Scientific Research, Jazan University, Saudi Arabia, through project number: (GSSRD-24).
