# Supplementary material for: Code-mixing unveiled: Enhancing the hate speech detection in Arabic dialect tweets using machine learning models
Source: PLoS One. 2024 Jul 17;19(7):e0305657. doi: 10.1371/journal.pone.0305657 (PMC11253949; doi:10.1371/journal.pone.0305657)
Supplement: S1 Table — (PDF) [file pone.0305657.s001.pdf]

Hate Speech category	keywords
Racist	جيزاني، صلبي، طرش بحر, بقايا حجاج
Sexism	نسويات، مكانها المطبخ
Offensive	List of keywords known as offensive toward groups or individuals.
